# Self-reported patterns of impairments in mentalization, attachment, and psychopathology among clinically referred adolescents with and without borderline personality pathology

**DOI:** 10.1186/s40479-017-0055-7

**Published:** 2017-02-14

**Authors:** Sune Bo, Mickey Kongerslev

**Affiliations:** 1 Psychiatric Research Unit, Region Zealand Psychiatry, Slagelse, Denmark; 2 Department of Child and Adolescent Psychiatry, Region Zealand Psychiatry, Roskilde, Denmark; 3 Centre of Excellence on Personality Disorder, Region Zealand Psychiatry, Slagelse, Denmark; 4 Department of Psychology, University of Southern Denmark, Odense, Denmark

**Keywords:** Borderline personality disorder, Personality disorder, Reflective functioning, Mentalization, Attachment, Adolescence

## Abstract

**Background:**

Previous research, which primarily focused on adult samples, suggests that individuals with borderline personality disorder (BPD) display high levels of psychopathology, dysfunctional mentalization and problematic attachment to others. The current study investigated whether impairments in mentalization, attachment, and psychopathology are more severe in outpatient adolescents with BPD than in a clinical comparison group.

**Methods:**

Consecutive referrals to a child and adolescent psychiatric clinic were clinically assessed with a battery of self-report instruments to assess mentalization, attachment, and psychopathology. Specifically, in regard to BPD a self-report questionnaire was employed to decide if patients were classified into the BPD or the clinical comparison group. The main outcome variables of adolescents with a primary diagnosis of BPD were then compared with those of a clinical comparison group comprising patients receiving psychiatric diagnoses other than BPD.

**Results:**

Relative to the clinical group without BPD, and after controlling for sociodemographic variables, the BPD group displayed poorer mentalizing abilities, more problematic attachments to parents and peers, and higher self-reported levels of psychopathology.

**Conclusions:**

The results of this study suggest that BPD is a severe mental condition in adolescents and is characterized by poor mentalizing abilities, attachment problems and high levels of psychopathology compared to adolescents with psychiatric disorders other than BPD. Hence, clinicians should consider BPD when conducting diagnostic assessments, and evidence-based treatments for this vulnerable group should be developed.

## Background

In adult populations, personality disorders (PDs) in general and borderline personality disorder (BPD) in particular are related to significant impairments in general functioning when compared to subjects without PD diagnoses and those with other mental disorders [1]. Specifically, evidence suggests that adults with PD exhibit poorer social and interpersonal functioning, are less likely to prospectively maintain an occupation, and report less life-satisfaction compared to people without PD [2–4]. Regarding adolescents, longitudinal studies show that early maladaptive and pathological personality features predict later social and functional impairments (i.e., failure to complete school, alcohol and drug dependence, and hazardous and antisocial behaviors) [5–10].

Additionally, studies report a high prevalence of PDs in both the general and clinical populations [11] and that these disorders are associated with excessive societal costs [12, 13]. The increasing attention given to and research conducted in the field of PDs in adult populations has encouraged the development of new and specialized treatments for adults with PDs, notably BPD, in the last two decades [14].

Historically, however, less attention has been given to PDs in childhood and adolescence [15]. Until recently, many clinicians and researchers did not acknowledge the existence of personality pathologies in adolescents [16–18]. Indeed, they did so despite the fact that, according to the *Diagnostic and Statistical Manual of Mental Disorders, Fifth Edition* (DSM-5) and its predecessors, PD diagnoses may be applied to adolescents when the individual’s particular maladaptive personality traits appear to be pervasive and persistent, are unlikely to be limited to a particular developmental state or to another mental disorder, and are present for 1 year or more [19].

Indeed, the available research suggests that PDs in adolescents younger than 18 years can be diagnosed as reliably and with as much validity as in adulthood and that the prevalence of PDs in adolescents in both the general population and clinical settings are comparable to those reported for adults [15, 17, 20, 21]. Developmental research suggests that PDs are moderately stable during adolescence [22] and are strongly related to childhood emotional difficulties and problematic behavior [23–25]. Furthermore, studies have indicated that delays in the diagnosis of PDs and the provision of interventions in adolescence can potentially result in devastating consequences and poor long-term prognoses [26–28].

Most theoretical and empirical developmental models of BPD either implicitly or explicitly assume that attachment problems or interpersonal trauma and difficulties are related to the later development of BPD. According to the mentalization-based model of BPD, the core pathology underlying BPD is associated with dysfunction in mentalization and insecure attachment patterns [29]. Mentalization refers to the ability to understand the self and others as intentional agents with minds [30]. Mentalizing is considered important for interpersonal functioning because it enables people to understand behavior in terms of mental states in regard to both the self and others [31]. Research has demonstrated that dysfunctions in mentalization are a core feature in patients with BPD [32], and based on many studies that link BPD and mentalizing dysfunctions, promising theories have been proposed that apply the mentalization-based model to explain the emergence of BPD in adolescents [33]. The mentalizing theory suggests that the capacity to mentalize is developed via the close relationship between a child and his or her primary caregiver and is dependent upon a secure attachment relationship [31] in which the primary caregiver adequately mirrors the child’s mental state. The mirroring process must be both *contingent* (e.g., fear is mirrored with fear and not joy) and *marked* (e.g., the mental state being mirrored must be similar but clearly different from that of the caregiver). Thus, a secure attachment relationship in which the caregiver benignly and accurately represents the child as an intentional agent with intentions, thoughts, and emotions underpins the development of the capacity to mentalize and secure the normative development of the child’s personality [34].

In contrast, the pathological trajectory leading to BPD is characterized by a caregiver who is unable to provide a secure attachment relationship, specifically defined by inadequate mirroring (i.e., un-marked and non-contingent; see [34] for details). In this case, because the caregiver is unable to mirror and represent the mind of the child, the child will display difficulties in understanding how actions and mental states are linked in the self and others [31]. In the mentalizing theory, the difficulties pertaining to insecure attachment relations and dysfunctional mentalizing, as explained above, are specifically believed to underlie BPD. This does not mean that BPD is the only psychiatric disorder characterized by dysfunctional mentalization and insecure attachment [31]. However, the mentalizing theory emphasizes these characteristics in particular as underlying BPD. Problematic attachment relations and dysfunctional mentalization have also been found in empirical studies in adolescents with BPD [35–38]. Recent empirical findings showed that problematic family functioning and low maternal care were predictors of BPD in adolescents [39], underscoring the role of attachment relationships between parents and children in the development of BPD in adolescents. Another recent study, displayed how BPD patients compared to non-BPD psychiatric controls and healthy controls, showed more dysfunctional emotion regulation, even when controlling for important sociodemographic and clinical variables [40]. In a community-dwelling study with Italian adolescents, findings showed that non-suicidal self-injury (NSSI) and emotional dysregulation are moderately related to BPD features in adolescents [41]. This finding was replicated by Kaees and colleagues [42] in adolescent inpatients with NSSI and suicide attempts (SA) and showed that dimensional borderline pathology was associated with NSSI and SA. In line with recent developmental theories explaining BPD (i.e., mentalisation-based theory), Sharp and colleagues [43] found that specifically hypermentalizing (i.e., ascribing intentions and beliefs to people where non is) mediated the relationship between attachment coherence and borderline pathology. In another study Ramos and colleagues [44] found, in a sample of 60 adolescents BPD patients, that attachment anxiety was positively related to internalizing psychopathology but negatively related to externalizing pathology. Furthermore, in a study examining the trajectories of borderline pathology and psychosocial functioning, results indicated that the development of BPD was significantly related to worsening in academic, social and mental health outcomes [45]. Finally, in a recent systematic review and meta-analysis, Winsper and colleagues [46] found that BPD in adolescents is related to the same aetiological and psychopathological issues as those found in adults with BPD.

Despite emerging theories on BPD in adolescents and research findings pointing to psychological dysfunctions in BPD, there still exist gabs in the research literature on BPD in adolescents. First, a variety of different clinical variables have been identified as pertaining to BPD, but rarely have they been investigated in the same study. Second, many studies have compared BPD groups to healthy controls but few have included a clinical non-BPD comparison group. Third, and specifically related to attachment, no studies have explored the quality of self-reported attachment in relation to both parents and peers in patients with and without BPD. Finally, and to the authors’ knowledge, no studies have been conducted as a naturalistic clinical comparison study in an ordinary child and adolescents’ psychiatric clinic, adding ecological validity to the findings. Hence, to the best of our knowledge, no studies have explored the differences between patients with and without borderline pathology in terms of attachment, mentalizing and psychopathology in a sample of adolescent psychiatric patients.

Thus, the aim of this study was to explore the patterns of impairment in an outpatient adolescent clinical sample diagnosed with BPD compared to those of a clinical group without PD but with other mental disorders. Specifically, we wanted to determine whether there was a difference between BPD and clinical comparison subjects with respect to attachment to peers and parents and mentalization. We also examined differences regarding the severity of psychopathology, self-harm and risk-taking behaviors, and depression. We hypothesized that the BPD group would display more problematic attachment relations, more mentalizing dysfunctions, a significantly higher level of psychopathology, more depressive features and more self-harm and risk-taking behaviours than the group without BPD. We also predicted that significant differences would be apparent from both the dimensional (number of borderline features) and categorical (meeting the criteria for a BPD diagnosis) perspectives.

## Methods

### Setting

This study was conducted at a Danish outpatient child and adolescent psychiatric clinic by a team that specifically focuses on handling adolescents, including those with PDs. This clinic specializes in the assessment and treatment of a broad range of mental health disorders in referred children aged 0 to 17 years in Region Zealand. Within this clinic, the team involved in this study specifically handles adolescents aged 13–18 years. Social authorities, general medical practitioners, psychiatrists, and school services can refer adolescents to this clinic. The staff at the clinic consists of experienced and qualified specialized psychiatrists, nurses, and clinical psychologists.

### Participants and procedure

All consecutive referrals to the team that focuses on adolescents within the child and adolescent psychiatric clinic from 2013 to 2015 were approached to participate in the study. Inclusion criteria were age between 13 and 18 years and Danish as the first language. In the BPD group, we only included patients with a BPD diagnosis as defined by a score of 66 or above on the Borderline Personality Feature Scale for Children (BPFS-C) [38]. The remaining patients (i.e., those not who did not receive a PD diagnosis) were included in the clinical comparison group.

All patients were seen at intake by at least two members of the staff for clinical and diagnostic assessments. These assessments also included interviews with family members and the collection of information from schools and social workers. The patients’ final clinical diagnoses were decided at weekly clinical conferences attended by both psychologists and child and adolescents psychiatrists. As a part of this study, all patients also filled out a battery of self-report questionnaires measuring borderline features, attachment, mentalization, externalizing and internalizing pathologies, self-harm and risk taking behaviors, and depression. These self-report questionnaires were administered within 2 weeks of the referral and were filled out by the adolescents before they knew which diagnosis they would receive at the clinic. The clinic staff was kept blinded to the data from the questionnaires until after the final decisions regarding the diagnoses were made. As no semi-structured PD interview was systematically administered to all patients, BPD diagnoses was decided based on the total score on the BPFS-C (see below). The total sample comprised 109 patients, 45 of whom received a diagnosis of BPD with reference to the BPFS-C. In the clinical comparison group, 25 participants were diagnosed with depression, 11 with attention-deficit/hyperactivity disorder (ADHD), 9 with anxiety disorders, 9 with other mixed disorders of conduct and emotions, 5 with pervasive developmental disorder, and 5 with conduct disorder. Nine patients were excluded because they were diagnosed with PDs other than BPD, and 17 of the referred patients never showed up to the initial clinical evaluation or moved during the assessment periode. Information on the sociodemographic characteristics of the total sample and stratified by groups is presented in Table 1.

**Table 1:** Sociodemographic characteristics of the total sample and by groups

Sociodemographic characteristics	Total sample (*N* = 109)	Borderline personality disorder group (*n* = 47)	Clinical comparison group (*n* = 62)
Age (Mean ± SD)	16.1 ± 1.1	15.9 ± 1.2	16.2 ± 1.1
Range in years	13–18	13–18	13–18
Gender			
Male	44 (40.4%)	14 (29.8%)	27 (43.5%)
Female	65 (59.6%)	33 (70.2%)	35 (56.5%)
Educational level			
Primary School	55 (50.5%)	28 (59.6%)	27 (43.5%)
High School	18 (16.5%)	4 (8.5%)	14 (22.6%)
Youth education	14 (12.8%)	3 (6.4%)	11 (17.7%)
None	22 (20.2%)	12 (25.5%)	10 (16.1%)
Upbringing			
Both parents	59 (54.1%)	24 (51.1%)	35 (56.5%)
Mother	44 (40.4%)	19 (40.4%)	25 (40.3%)
Father	2 (1.8%)	2 (4.3%)	0 (0%)
Fostercare	4 (3.7%)	2 (4.3%)	2 (3.2%)
Current living arrangements			
Parents	89 (81.7%)	37 (78.7%)	52 (83.9%)
Appartment	2 (1.8%)	2 (4.3%)	0 (0%)
Fostercare	18 (16.5%)	8 (17.0%)	10 (16.1%)
Civil Status			
Single	77 (61.5%)	29 (61.7%)	38 (61.3%)
In a relationship	42 (38.5%)	18 (38.3%)	24 (38.7%)
Job-status (beside school)			
In a job	31 (38.4%)	12 (25.5%)	19 (30.6%)
Not in a job	78 (71.6%)	35 (74.5%)	43 (69.4%)

### Measures

#### BPFS-C [38]

The BPFS-C assesses borderline personality traits dimensionally and was adapted from the Borderline Scale of the Personality Assessment Inventory (PAI; [47]) for use with children and adolescents. This scale is composed of 24 items, which are summed to yield a total score after four of the items are reverse scored. Each item is scored on a five-point Likert scale ranging from 1 (not at all true) to 5 (always true). Higher scores indicate greater levels of borderline personality features. Crick and colleagues [38] demonstrated high internal consistency and established evidence of the scale’s construct validity. Sharp and colleagues provide further evidence supporting its criterion validity, cross-informant concordance, and concurrent validity [48]. Chang and colleagues [49] found that the optimal cut-off score for discriminating BPD among adolescent inpatients using the BPFS-C was 66. The area under the curve (AUC) was .931, indicating high accuracy of the BPFS-C instrument in regard to the gold standard semi-structured interview. The BPFS-C was included in the present study to assess borderline pathology both categorically and dimensionally. In the current study, Cronbach’s α was 0.90.

#### Youth self-report (YSR) [50]

The YSR is a widely used questionnaire that measures a broad range of psychopathologies in young people aged 11 to 18 years. It includes 112 problem items, each of which can be rated 0 (not true), 1 (somewhat or sometimes true) or 2 (very true or often true). The YSR has shown excellent psychometric properties and good correspondence with specific DSM diagnostic categories [51, 52]. In the present study, we used the two broad subscales of Internalizing and Externalizing Psychopathologies. The Internalizing scale is composed of the Anxious/Depressed, Withdrawn/Depressed, and Somatic Complaints scales, whereas the Externalizing scale includes two subscales: Aggressive Behavior and Rule-breaking Behavior. The Cronbach’s α in this study was 0.95.

#### Beck depression inventory for youth (BDI-Y) [53]

The BDI-Y is used to assess depressive features in children and adolescents aged 7 to 18 years old. This test consists of 20 questions about depressive symptomatology within the past 14 days, each of which is rated from 0 (never) to 3 (always). The BDI-Y is widely used and has demonstrated adequate psychometric properties [54]. In the current study, Cronbach’s α was 0.94.

#### Risk-taking and self-harm inventory for adolescents (RTSHI-A) [55]

The RTSHI-A is composed of 38 items adapted from the adult Self Harm Inventory (SHI, [56]) and is used to assess risk-taking and self-harming behaviors in children and adolescents. This measure requires the adolescent to rate the frequency with which he or she has engaged in self-harm or risk-taking behaviors using a four-point Likert scale. The RTSHI has been shown to have acceptable psychometric properties [55]. Cronbach’s alpha in this study was 0.88.

#### Inventory of parent and peer attachment - revised (IPPA-R) [57]

The IPPA-R is a reliable and valid 53-item self-report questionnaire that measures attachment in adolescence. This instrument is composed of two scales that measure attachment to parents and peers. For each of the 28 items assessing parent attachment and 25 items assessing peer attachment, respondents are required to rate the degree to which each item is true for them on a five-point scale that ranges from ‘Almost always or always true’ to ‘Almost never or never true’. Higher scale scores indicate more problematic attachment relations to parents and peers. In this study, Cronbach’s α = 0.91.

#### Reflective function questionnaire for youth (RFQY) [58]

The RFQY is a 46-item self-report questionnaire designed to measure the general capacity for reflective function or mentalizing. Each item is rated on a 6-point Likert scale ranging from Strongly Disagree to Strongly Agree. A total scale score can be derived by summing the individual item scores. High total scores indicate higher capacities for mentalizing. The RFQY demonstrated good psychometric properties, including construct validity, in a recent psychometric study [59]. Cronbach’s alpha in the current study was 0.88.

Danish-translated versions of these instruments were used in this study.

### Statistical analysis

SPSS version 23 for MAC was used to conduct all of the statistical analyses. Prior to testing the hypothesis, we performed preliminary analyses to determine the means, standard deviations and ranges for all variables included in the study. Then, independent t-tests were conducted to identify significant differences between the BPD group and the clinical comparison group in attachment (IPPA-Peer and IPPA-Parent), mentalization (RFQ:YV), borderline pathology (BPFS-C), depression (BDI-Y), self-harm and risk-taking behaviors (RTSHIA), and internalizing and externalizing pathologies (YSR-internalizing and YSR-externalizing scales). Next, Pearson correlational analysis was used to explore the bivariate relationships between all variables in the study and to elucidate the dimensional relationship between borderline pathology and attachment and mentalization. Finally, we conducted a multivariate analysis of covariance (MANCOVA); the grouping variable was BPD versus clinical comparison subjects without BPD, and the dependent variables were attachment (IPPA-Peer and IPPA-Parent), mentalization (RFQ:YV), borderline pathology (BPFS-C), depression (BDI-Y), self-harm and risk-taking behaviors (RTSHIA), and internalizing and externalizing pathologies (YSR-internalizing and YSR-externalizing scales). The MANCOVA analysis was followed by a separate analysis of variance (ANOVA) for each dependent variable [60]. The datasets analyzed in the current study are available from the corresponding author upon request.

## Results

Table 2 shows that the independent *t*-test revealed significant differences between the BPD and clinical comparison group for all variables. Thus, relative to the clinical comparison group, the BPD group displayed higher levels of self-reported BPD features, internalizing and externalizing psychopathologies, depressive symptomatology, impulsivity and self-harm and poorer mentalizing capacity and attachment to parents and peers. When examining the borderline pathology dimensionally, we found the same pattern. The bivariate correlations indicate that more borderline pathology is correlated with increased dysfunctional mentalization, problematic attachment relations to both parents and peers, and psychopathology, including depression and self-harm (see Table 3).

**Table 2:** Comparison of clinical characteristics among patient groups

Clinical Measures	All (*N* = 109)	Borderline Personality disorder group (*n* = 47)	Clinical comparison group (*n* = 62)
	Mean (SD)/%	Mean (SD)	Mean (SD)
BPDFS-C (score)	69.7 (16.8)	86.6 (9.6)^*^	56.8 (6.8)^*^
YSR: Externalizing	22.0 (10.5)	29.1 (8.0)^*^	16.6 (8.8)^*^
YSR: Internalizing	29.5 (12.3)	38.8 (8.3)^*^	22.5 (10.0)^*^
RFQY	8.2 (1.3)	7.2 (1.0)^*^	9.0 (0.9)^*^
BDI-Y	49.2 (11.9)	56.8 (9.8)^*^	43.4 (9.6)^*^
IPPA-Peer	47.4 (8.5)	54.6 (4.2)^*^	42.2 (7.0)^*^
IPPA-Parent	54.3 (9.6)	59.2 (7.6)^*^	50.3 (9.6)^*^
RTSHIA	59.4 (15.3)	66.7 (14.7)^*^	53.8 (13.4)^*^

Note. *BPFS-C* The Borderline Personality Features Scale for Children, *YSR* The Youth Self-Report, *RFQ-Y* Reflective Function Questionnaire for Youth, *IPPA* inventory of parent and peer attachment, *RTSHI* risk-taking and self-harm inventory, *BDI-Y* Beck Depression Inventory for Youth

^*^Statistically significant difference between the borderline personality disorder group versus clinical comparison group, *p < .*001

**Table 3:** Bivariate correlations between main study variables

Variables	BPFS-C Total	RFQ-Y	IPPA-Parent	IPPA-Peer	YSR Total	YSR-Externalising	YSR-Internalising	BDI-Y
BPFS-C Total	1.00							
RFQ-Y	-.72^**^ [−.80, −.62]	1.00						
IPPA-Parent	.50^**^ [.37, .62]	-.38^**^[−.51, −.22]	1.00					
IPPA-Peer	.65^**^ [.54, .75]	-.58^**^ [−.45, −.75]	.38^**^[.22, .54]	1.00				
YSR Total	.83^**^ [.79, .88]	-.75^**^ [−.81, −.67]	.54^**^ [.40, .65]	.66^**^ [.56, .76]	1.00			
YSR-Externalising	.68^**^ [.55, .80]	-.65^**^ [−.74, −.54]	.44^**^ [.24, .61]	.48^**^ [.33, .63]	.80^**^ [.72, .86]	1.00		
YSR-Internalising	.73^**^ [.64, .81]	-.63^**^ [−.71, −.53]	.48^**^ [.31, .61]	.60^**^ [.48, .70]	.85^**^ [.78, .90]	.43^**^ [.24, .61]	1.00	
BDI-Y	.63^**^ [.48, .75]	-.53^**^ [−.66, −.37]	.46^**^ [.29, .61]	.58^**^ [.44, .71]	.71^**^ [.56, .81]	.39^**^ [.19, .58]	.79^**^ [.69, .86]	1.00

Note: *BPFS-C* The Borderline Personality Features Scale for Children, *YSR* The Youth Self-Report, *RFQ-Y* Reflective Function Questionnaire for Youth, *IPPA* inventory of parent and peer attachment, *RTSHI* risk-taking and self-harm inventory, *BDI-Y* Beck Depression Inventory for Youth

Bias corrected and accelerated bootstrap 95% CIs are reported in square brackets

### Clinical differences between the BPD and clinical comparison groups

MANCOVA was used to test for differences between the BPD and clinical comparison groups in attachment, mentalization, borderline features, externalizing and internalizing pathologies, depression, impulsivity and self-harm while controlling for age, gender, educational level, and living status. According to the Pillai’s trace test, the results revealed significant differences between the BPD and clinical comparison groups: *V* = 0.80, *F*(8,96) = 48.1, *p* < 0.001 (Table 4). Note that none of the covariates were significantly related to BPD. Box’s M indicated that the assumption of equality of covariance matrices for the MANCOVA was not violated (*p* = 0.06).

**Table 4:** MANCOVA analysis of BPD versus clinical comparison subjects as a function of attachment, mentalizing, borderline features, emotional dysregulation, externalizing and internalizing pathology, depression and risk taking and self-harm after controlling for sociodemographic variables

MANOCOVA Test: Multivariate test				
Effect	Pillai’s trace *V*	F	df	*p*
Intercept	0.68	23.41	8	0.001
Age	0.08	1.04	8	0.41
Gender	0.05	0.58	8	0.80
Educational Level	0.11	1.52	8	0.16
Living status	0.10	1.31	8	0.25
Grouping variable (BPD vs. clinical comparisons)	0.80	48.14	8	0.001

As shown in Table 5, separate univariate ANCOVAs performed for the outcome variables revealed a significant effect between the BPD and clinical comparison groups on all variables. Thus, significant differences between the two groups were found for attachment, mentalizing abilities, borderline features, depressive symptomatology, externalizing and internalizing pathologies, risk-taking behavior and self-harm.

**Table 5:** ANCOVA analysis of group differences between the borderline and clinical comparison subjects as a function of attachment, mentalizing, internalizing and externalizing psychopathology, depression, borderline features, and impulsivity and self-harm

ANCOVA test: Test of Between-subjects Effect				
Dependent variable	Type III Sum of Squares	df	F	*p*
RTSHIA (impulsivity and self-harm)	3942.3	5	20.6	0.001
BDI-Y (depression)	4256.6	5	45.7	0.001
RFQ-Y (mentalizing)	81.7	5	83.8	0.001
YSR-internalizing (internalizing pathology)	6609.3	5	77.0	0.001
YSR-externalizing (externalizing pathology)	3511.5	5	51.8	0.001
IPPA-Peer (attachment to peers)	3690.4	5	98.7	0.001
IPPA-Parent (attachment to parents)	1739.7	5	22.8	0.001
BPFS-C (Borderline features)	22549.0	5	337.9	0.001

Note: *BPFS-C* The Borderline Personality Features Scale for Children, *YSR* The Youth Self-Report, *RFQ-Y* reflective function questionnaire for youth, *IPPA* inventory of parent and peer attachment, *RTSHI* risk-taking and self-harm inventory, *BDI-Y* Beck Depression Inventory for Youth

## Discussion

In this study, we explored the differences in mentalization, attachment, and psychopathology between adolescents with BPD and clinical comparison subjects both dimensionally and categorically. As predicted, more severe borderline pathologies were correlated with poorer mentalizing abilities, problematic attachment relations to parents and peers, and higher levels of risk-taking behaviors, self-harm, depressive symptomatology, and internalizing and externalizing psychopathologies. When exploring the differences between groups, using the optimal cut-off for the BPFS-C (a total score of 66 or above) to categorize patients into the BPD or clinical comparison group, we found the same results. In the BPD group, we observed significantly more mentalizing dysfunctions, more problematic attachment relations to both peers and parents, and more severe levels of psychopathology, including depression and a greater propensity for self-harm.

Taken together, these findings suggest that adolescents diagnosed with BPD face a wide range of severe and complex impairments in their mentalizing abilities, difficulties with attachment, and high levels of both internalizing and externalizing psychopathologies. Thus, in adolescents, BPD is a severe disorder that is associated with both poor psychological well-being and high treatment needs. The finding that participants diagnosed with BPD display high levels of both internalizing and externalizing psychopathologies has also been observed in a large population-based sample of adults in the US [61] and in hospitalized adolescents [62]. Indeed, complex co-occurrence across the spectra of internalizing and externalizing psychopathologies appears to be a fairly characteristic feature of BPD in adolescence and adulthood and may indicate of a common susceptibility to distress, mental pain, and externalization [61, 63]. The results reflecting dysfunctional mentalization and problematic attachment relations in the BPD group are in good agreement with the mentalization-based model for BPD [31] and empirical findings showing that the core pathology of BPD in adolescents is related to dysfunctional mentalization and problematic attachment relations [29, 32, 33, 35, 36]. Thus, the findings of this study support the hypothesis that problematic attachment relations to both parents and peers and dysfunctional mentalization may be core features in the understanding and development of BPD [36].

Regarding BPD in adolescents, theories suggest that incapacities in mentalizing functioning are specifically characterized by a tendency to over-attribute intentions, beliefs, and wishes to people in situations where there is no proof supporting such attributions [33]. This form of dysfunctional mentalizing is termed *hypermentalizing* and can potentially cause substantial interpersonal difficulties [29]. The over-attribution of intentions to other people in social situations can easily lead to misunderstandings that can cause conflicts and problematic relationships. If left out of social interpersonal relationships, the adolescent can then become isolated from the valuable social and cultural knowledge (epistemic isolation) necessary for normative development [31]. The results of this study do not specifically indicate a hypermentalizing profile for the BPD group because we were unable to detect such dysfunctions using the instruments included in this work. However, the results are in line with the general mentalization-based theory of BPD, which identifies profound mentalizing dysfunctions as especially characteristic for and underlying BPD symptomatology [64].

Another noteworthy finding was the differential discriminative abilities between the BPD and clinical control groups in terms of parental and peer attachments. Although the empirical literature generally shows evidence that supports links between attachment difficulties and concurrent and prospective associations with psychopathology and BPD, there is a paucity of data on the potential differential effects of parent versus peer attachment problems on the development of psychopathology in general and BPD specifically [65, 66]. Additionally, this result appears to be consistent with both diagnostic classifications and theoretical approaches, including empirical research, that highlight pervasive interpersonal difficulties and dysfunctions as core features of PDs, including BPD [67–70]. Thus, this study indicates that BPD in adolescents is related to substantial attachment problems regarding both parents and peers relative to a clinical comparison group. These findings suggest that adolescents with BPD face great potential risks regarding their normative developmental processes [31, 64] because these individuals must struggle to establish the stable relationships with both parents and peers that are necessary for healthy development.

### Limitations

This study has several limitations. First, the diagnostic assessments were based on clinical interviews, and systematic standardized and structured clinical instruments were not always used. This diagnostic procedure is subject to a range of psychometric issues, including a high risk of overlooking psychopathology and poor inter-rater reliability [71]. Additionally, most of the variables of interest in this study were self-reported, and self-reporting is known to be subject to many potential psychometric issues, such as biased responding. For this reason, future studies should include other types of measures and measurement methods when further investigating dysfunction and psychopathology in BPD.

Another limitation relates to the cross-sectional nature of the study design, which does not allow for inferences about causal relationships and issues relating to the longitudinal relationships between variables. This issue should be addressed in future studies. Furthermore, whether our results are generalizable to populations with more or less severe levels of pathology, such as outpatients or community samples, is unknown. Finally, the small sample size did not allow us to robustly test the potential effects of gender or age.

## Conclusions

Despite the aforementioned limitations, our study highlights that, in a clinical sample of adolescents, BPD is associated with significantly more severe self-reported mentalization dysfunctions, attachment problems and psychopathology relative to a clinical comparison group without BPD. The results also suggested that poor mentalizing abilities and problematic attachments to peers and parents characterized the BPD group compared to the clinical comparison group. This finding is in line with the recently developed mentalization-based theory for BPD. The potentially differential role of attachment to peers in adolescents with BPD compared to attachment to parents or other significant others is an important area that should be addressed in future research.

Taken together, the findings of the present study highlight the importance of clinicians being aware of BPD when assessing adolescents and demonstrate that poor mentalizing abilities and interpersonal dysfunctions may be important treatment targets in addition to the more behavioral manifestations of the BPD syndrome, such as self-harm. Fortunately, new and promising psychosocial treatments targeting BPD in adolescents are being developed and will hopefully become more broadly available to these vulnerable young people and their families in the near future [16, 35, 72–74]. The clinical recognition of BPD and the availability of evidence-based treatments for this debilitating disorder are both crucial to our ability to help these young people and their families.

## Abbreviations

ADHD: Attention deficit hyperactivity disorder
ANCOVA: Analysis of covariance
BDI-Y: Beck depression inventory for youth
BPD: Borderline personality disorder
BPFS-C: Borderline personality features scale for children
CCG: Clinical comparison group
DSM: Diagnostic and statistical manual of mental disorders
IPPA-R: Inventory of Parent and Peer Attachment – Revised
MANCOVA: Multivariate analysis of covariance
PD: Personality Disorder
RFQY: Reflective function questionnaire for youth
RTSHI-A: Risk-Taking and self-harm inventory for adolescents
SPSS: Statistical program software package
YSR: Youth self-report
